# Prolapse of Rectum in Child One Year Old

**Published:** 1894-08

**Authors:** Ira W. Porter

**Affiliations:** Sanford, Fla.


### PROLAPSE OF RECTUM IN CHILD ONE YEAR OLD.

BY IRA W. PORTER, M.D., SANFORD, FLA.

Recently I was called in to attend a case of prolapse of the rectum The patient was a baby girl, Cuban, one month old. The prolapse was of ten days' standing.

The father could not speak English very well and the mother not at all. Through an interpreter I learned that the child had been having hemorrhages from the bowels for two days, during which the rectum prolapsed. The hemorrhages continued for a few days longer, but had been stopped by medicine obtained through a druggist.

On examination, I found the rectum protruding about one inch and a half or two inches. The protrusion was dark red and angry looking. There was the characteristic depression at the apex which proved to be the lumen of the gut. The base of the protrusion was constricted by a dark ring of mucous membrane corresponding in position to the anus. The protrusion was very sensitive to touch. Placing my finger on it excited peristaltic movements, which caused the bowel to protrude farther.

I tried reduction by taxis and compression, but without avail. The child, in my opinion, was too young to anesthetize with chloroform or ether, so my only recourse was to local applications. I debated in my mind whether it were safe to anesthetize the gut with cocaine and then reduce by taxis. While I was debating, I prescribed an ointment of oil of hamamelis one drachm, tannic acid five grains, vaseline one ounce, and directed that some of the ointment be spread on a piece of absorbent cotton and applied to the gut by a compress. This to be done twice a day.

The next day the protrusion had receded nearly two-thirds of its length. I ordered the application to be continued, and the third day thereafter I found my patient well. During the treatment there was no hemorrhage, and the bowels acted normally. There has been no return of the prolapse.

